# Block Copolymer-Based Membranes for Vanadium Redox Flow Batteries: Synthesis, Characterization, and Performance

**DOI:** 10.1021/acsapm.4c01262

**Published:** 2024-07-29

**Authors:** Sydonne Swaby, Diego Monzón, Nieves Ureña, José Vivo Vilches, Jean-Yves Sanchez, Cristina Iojoiu, Alejandro Várez, María Teresa Pérez-Prior, Belén Levenfeld

**Affiliations:** † Departamento de Ciencia e Ingeniería de Materiales e Ingeniería Química, IAAB, Universidad Carlos III de Madrid, Avda. Universidad, 30, 28911 Leganés, Madrid, Spain; ‡ LEPMI, University Grenoble Alpes, 38000 Grenoble, France; § CNRS, LEPMI, 38000 Grenoble, France

**Keywords:** block copolymer, polysulfone, polyphenylsulfone, proton exchange membrane, impedance spectroscopy, vanadium redox flow batteries

## Abstract

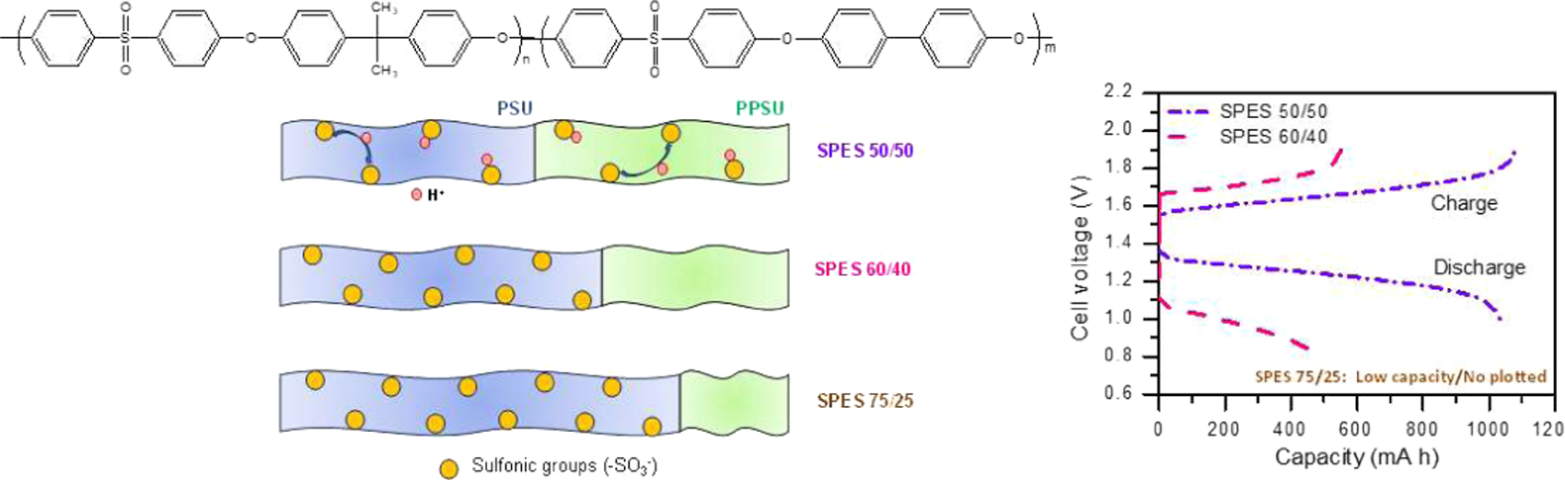

Nonfluorinated polymers have been widely proposed to replace Nafion as raw materials for redox flow battery ion-exchange membranes. Hereby, block copolymers based on polysulfone (PSU) and polyphenylsulfone (PPSU) are synthesized and employed as precursors of membranes for vanadium redox flow batteries. A series of copolymers with varying molar proportions of PSU (75/25, 60/40, 50/50 mol %) were prepared. The 60/40 and 75/25 copolymers exhibit concentrated sulfonic groups predominantly in the PSU unit, favoring the formation of hydrophobic and hydrophilic domains. The 50/50 copolymer presents a balanced degree of sulfonation between the two units, leading to a homogeneous distribution of sulfonic groups. An *ex situ* study of these materials comprising vanadium ion permeability and chemical and mechanical stability was performed. The best performance is achieved with 50/50 membranes, which exhibited performance comparable to commercial Nafion membranes. These results signify a promising breakthrough in the pursuit of high-performance, sustainable membranes for next-generation VRFBs.

## 1 Introduction

The development of energy storage systems is growing by leaps and bounds, as it is considered one of the key solutions to effectively integrate energy from renewable energy sources such as solar or wind power into the electrical grid. In 2021, renewable electricity generation rose by almost 7%, a record 522 TWh increase, with wind and solar photovoltaic (PV) technologies together accounting for almost 90% of this growth.^1^ Among the various energy storage systems, redox flow batteries (RFBs) are postulated as candidates.^2−4^ RFBs exhibit a flexible modular design, good scalability, moderate maintenance costs, and long-life cycling.

Vanadium redox flow batteries (VRFBs) are a mature technology that has been widely studied. They show better cell efficiency and long-term stability than other RFBs.^4^ A VRFB is composed of VO_2_^+^/VO^2+^ and V^3+^/V^2+^ redox couples (in strong acid media) as the catholyte and anolyte, respectively, placed in two tanks. These active species are pumped into the flow cell, where electron transfer occurs on the electrode surface. Inside the cell, the ion-exchange membrane (IEM) acts as a separator to prevent the mixing of vanadium ions, a nondesired process known as crossover, whereas it allows the ion-conduction to balance the overall reaction. The ideal membrane should have high ionic conductivity, no electrical conductivity, low crossover of active species, high chemical stability, and low cost.^5^ So, in order to achieve these requirements, cation and anion-exchange membranes are being widely studied.^6−8^

Currently, commercial perfluorinated IEMs such as Nafion are the most widely used mainly due to their good ionic conductivity and marked chemical stability in acidic and oxidizing solutions as the ones used in VRFBs (vanadium ions in H_2_SO_4_).^9,10^ However, Nafion shows the high cost and undesirable crossover of active species, which hampers its application in VRFBs.

As an alternative to Nafion, sulfonated aryl backbone polymers such as sulfonated polyether ether ketone (SPEEK) or sulfonated polyether sulfone (SPES) are considered promising membrane materials mainly due to their reasonable price, good thermal and mechanical stability, and high ionic conductivity.^5,11^ The latter can be significantly improved by the incorporation of sulfonic groups. However, a high degree of sulfonation (DS) leads to high water uptake (WU), which is closely related to swelling, which reduces the mechanical stability of the membrane and, in some cases, causes its dissolution in aqueous media.^12^ This behavior becomes even more relevant in VRFBs,^13,14^ since the extremely high vanadium ion permeability can lead to lower VRFB performance. Some of the materials developed to overcome these drawbacks are shown as follows: (i) membranes based on cross-linked polymers in which the new networks formed improve the chemical and mechanical stability of the resulting membranes since they can reduce the attacks from oxidizing agents by suppressing the membrane swelling;^11,15^ (ii) hybrid membranes composed of a polymer matrix and organic/inorganic fillers between polymeric chains whose interactions are established that improve their proton conductivity, mechanical stability, and vanadium permeability,^16−18^ and (iii) membranes based on multiblock copolymers with well-defined hydrophobic and hydrophilic blocks which exhibit high stability thanks to the control of water absorption capacity.^19^

The main advantage of multiblock copolymers is associated with their robust structure, which can incorporate a high percentage of sulfonic groups without reducing their dimensional stability. This behavior could help to design new membranes with the VRFB requirements, *i.e.*, high proton conductivity, low permeability to vanadium species, and good chemical stability under VRFB operating conditions. Regarding the last point, the effect that the hydrophobic block has on the chemical stability of membranes has been investigated.^20^ The main results extracted from this study reveal that the nature of the hydrophobic block does not substantially modify the physical, electrochemical, and morphological properties of the membrane, but it does influence its chemical stability evaluated by means of an *in situ* VRFB cell test. Specifically, IEMs based on the styrene–divinylbenzene copolymer contain hydrophilic channels and hydrophobic blocks, which are responsible for their proton conductivity and mechanical stability, respectively.^21^ As a result, membranes showing a low permeability to vanadium ionic species have been prepared. Carboxyl-containing polyimide (PID) grafting sulfonated poly(vinyl alcohol) (SPVA) copolymer membranes have also shown high chemical stability because of the covalent bonds established between PID and SPVA, as well as their highly dispersed microphase-separated structure.^22^

In the present work, a series of IEMs based on SPES copolymers have been synthesized and characterized to evaluate their behavior as separators in a VRFB. The following polysulfone/polyphenylsulfone (PSU/PPSU) ratios (in mol %) have been chosen to prepare the SPES copolymers: 50/50, 60/40, and 75/25. The percentage of sulfonic groups in all of the studied membranes is high enough to ensure that their viability is as efficient as that of ionic conductors. In addition, they show good mechanical behavior, even at elevated degrees of sulfonation. After characterizing surface morphology, mechanical properties, chemical composition, permeability, and conductivity of the prepared membranes, the more suitable candidate was selected for VRFB tests.

## 2 Experimental Section

### 2.1 Materials

4,4′-Dihydroxybiphenyl (BP), 4,4′-difluoro-diphenylsulfone (DFDPS), and 4,4′-isopropylidenediphenol (BPA) were purchased from Alfa Aesar. They were recrystallized in isopropyl alcohol before being used. Carbon felt (6.35 mm, 99%, Alfa Aesar) was thermally treated in air prior to use (400 °C and 30 h). Nafion N-115 membrane, 0.125 mm thick and 0.90 mequiv g^–1^ exchange capacity, was purchased from Thermo Scientific. Vanadium(IV) sulfate oxide hydrate (VOSO_4_·H_2_O, 99.9%), trimethylsilyl chlorosulfonate (TMSCS, 99.0%), graphite, chloroform-*d* (CDCl_3_-*d*, 99.9% D), toluene (99.8%), sulfuric acid (H_2_SO_4_, 97%), dimethylformamide (DMF, ≥99.8%), and 1,2-dichloroethane (DCE, 98.9%) were supplied by Sigma-Aldrich. *N*,*N*-Dimethylacetamide (DMAc, 99.0%), dimethyl sulfoxide-*d*_6_ (DMSO-*d*_6_, 99.9%), and potassium carbonate (K_2_CO_3_, ≥99.0%) were purchased from Acros Organics and used as received.

### 2.2 Synthesis of Multiblock Copolymers and Preparation of Membranes

**Scheme 1:**
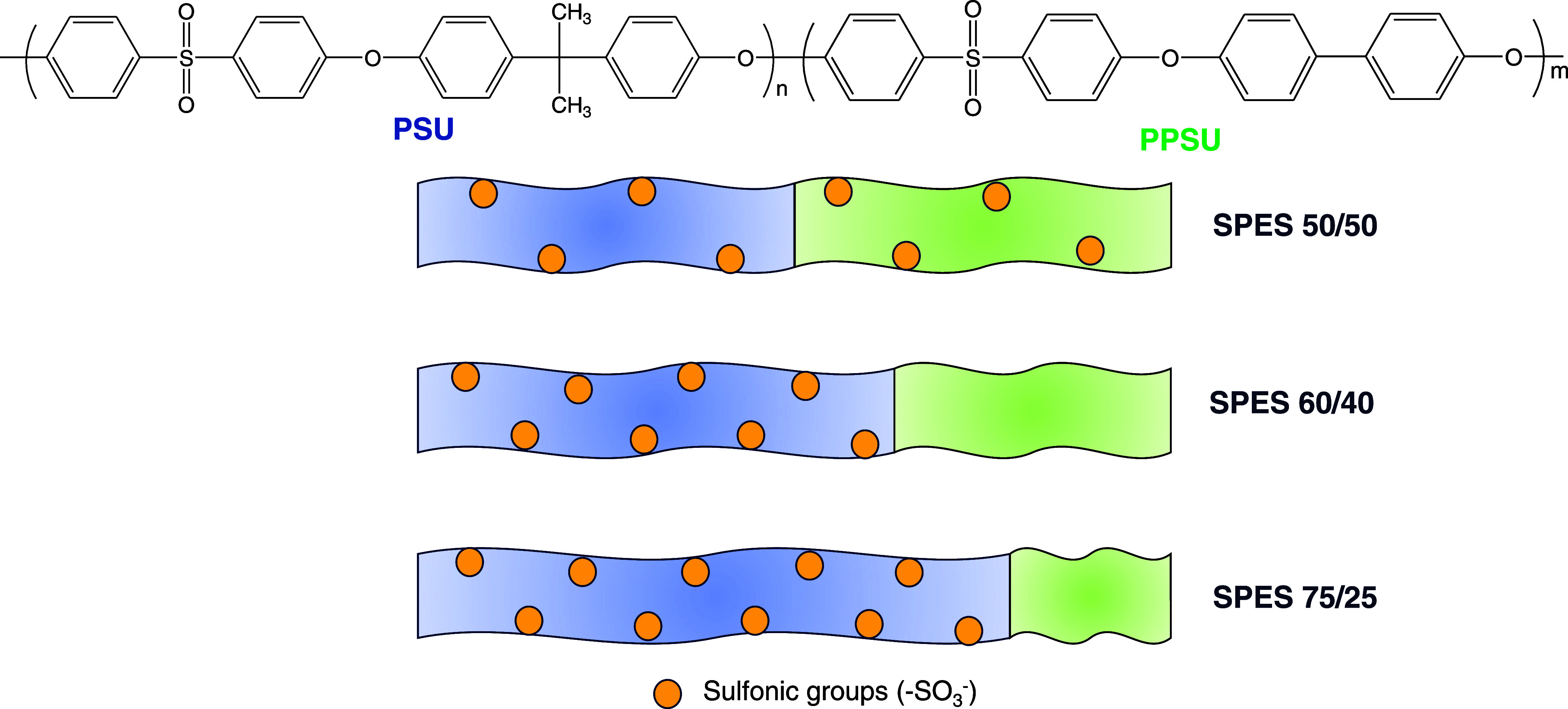
Chemical Structure of SPES 50/50, 60/40, and 75/25 for a Sulfonating Agent/Copolymer Ratio of 1/9

Multiblock copolymers based on PSU and PPSU blocks (PES) with three PSU/PPSU (in mol %) ratios (50/50, 60/40, and 75/25) were synthesized through a polycondensation in “one pot two-steps” from BP, DFDPS, and BPA monomers as it was previously reported by Swaby et al.^23^ (Figure S1). The variation obtained in the blocks of PSU and PPSU was controlled by the ratio of molecular weight of the blocks (1, 1.5, and 3 for PES 50/50 (5000/5000 g mol^–1^), PES 60/40 (7500/5000) g mol^–1^, and PES 75/25 (7500/2500) g mol^–1^, respectively (Scheme 1)). Sulfonated copolymers (SPES) were obtained by using a 1/9 sulfonating agent (TMSCS)/copolymer ratio, as shown in Figure S1.

Membranes based on SPES were prepared by casting. A solution of SPES (1.2 g) in DMF (22 mL) was deposited on a Petri dish in an oven at 60 °C over 48 h. The thickness of the resulting membranes was around 50 μm. Before their use, SPES membranes were immersed in a 2 M H_2_SO_4_ solution for 24 h at 30 °C to replace Na^+^ with H^+^. Next, they were rinsed with deionized water several times and stored in a 2 M H_2_SO_4_ solution at room temperature (RT).

### 2.3 Membrane Characterization

#### 2.3.1 ^1^H NMR

The chemical structure of copolymers was elucidated by means of ^1^H NMR in a Bruker Avance DPX-300 (300 MHz) spectrometer. Deuterated solvents such as CDCl_3_-*d* and DMSO-*d*_6_ were used to prepare the samples. Tetramethylsilane (TMS) was used as the internal reference solvent.

#### 2.3.2 Field Emission Scanning Electron Microscopy (FE-SEM)

Field emission scanning electron microscopy (FEI TENEO equipped with an EDS-EDAX) with an accelerating voltage of 15 kV and low vacuum was used to analyze the membrane morphology.

#### 2.3.3 Water Uptake and Swelling Degree

Considering the acidic environment in the cell, the water uptake (WU%) of the membranes was determined in a 2 M H_2_SO_4_ aqueous solution (WU_ac_%). The weight of fully hydrated membranes was measured after this treatment for 48 h (*W*_wet_). This parameter was measured following the same experimental protocol used for the determination of WU%, *i.e.*, membranes were immersed in an aqueous solution of 2 M H_2_SO_4_ for 48 h at these two temperatures, 30 and 60 °C. Then, the membrane was dried under vacuum at 60 °C for 24 h, and the weight was measured (*W*_dry_). The WU_ac_% was calculated by using the following equation
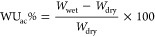
1The swelling ratio (SD) of the membranes was determined to study the dimensional change of the dried membranes (10 mm × 10 mm) before and after being soaked in a 2 M H_2_SO_4_ aqueous solution for 48 h at 30 °C. The SD was calculated taking into account both thickness (SD_Thickness_%) and area (SD_Area_%) variations using the following equations
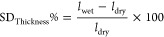
2
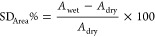
3

#### 2.3.4 Vanadium Ion Permeability

The vanadium ion permeability of the IEMs was tested in a homemade cell containing two compartments separated by the membrane (Figure S3). A 1 M VOSO_4_ solution in 2 M H_2_SO_4_ (40 mL) was placed in compartment A, whereas compartment B was filled with a 1 M MgSO_4_ solution in 2 M H_2_SO_4_ (40 mL) to equilibrate the osmotic pressure. Both solutions were kept under stirring at room temperature. Aliquots of the solution in compartment B were taken at different times to determine the variation of the concentration of VO^2+^ by means of UV–vis spectrophotometry in Jasco V-650 equipment. The permeability (*P*) was calculated by using the following equation^24,25^
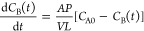
4where *C*_B_(*t*) is the concentration of VO^2+^ ions in compartment B as a function of time, and *C*_A0_ is the initial concentration of VO^2+^ in compartment A. The membrane area (1.21 cm^2^) and thickness (50 μm) are denoted as *A* and *L*, respectively. *V* is the volume of the solutions contained in each compartment and *P* is the VO^2+^ permeability, which was obtained from the slope of the ln(*C*_A0_ – 2*C*_B_(*t*)) – ln(*C*_A0_) *vst* plot.

#### 2.3.5 Mechanical Properties

A dynamo mechanical analysis (DMA) with DMA Q800 equipment (TA Instruments) was used to analyze the mechanical behavior of SPES-based membranes. Samples with dimensions of 2.5 × 10 mm^2^ (wide) were analyzed in tensile test mode by using an initial static force of 0.15 N. A controlled force mode with a ramp force of 0.3 N min^–1^ (until 18.0 N) was used to perform stress–strain tests at 30 °C.

#### 2.3.6 Chemical Stability

The membrane’s accelerated chemical stability was studied in an acidic 1 M VO_2_^+^ solution and 2 M H_2_SO_4_ at 40 °C. These experimental conditions are more aggressive than the environment reached in the redox flow batteries at a fully charged state in half cell. The presence of vanadium(V) species in this medium can oxidize the hydrocarbon chains of the copolymers,^26^ and this undesired reaction can be easily followed by the formation of vanadium(IV) ions by UV–vis spectrophotometry.

The membrane was immersed in a sealed bottle containing a 1 M VO_2_^+^ solution in 2 M H_2_SO_4_ at 40 °C for 56 days. Aliquots of the solutions were taken at different times and diluted into 2 M H_2_SO_4_ before measuring the absorbance of the samples at 760 nm, where the spectrum for VO^2+^ species shows an absorption maximum. The VO^2+^ concentration in the VO_2_^+^ solution was obtained from the absorbance *vs* concentration calibration curve obtained for VO^2+^ solutions.^11,25,27,28^

The weight loss and reduction of VO_2_^+^ to VO^2+^ were calculated through eqs 5 and 6, respectively, as follows
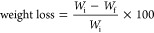
5
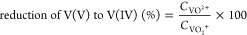
6where *W*_i_ and *W*_f_ are the membrane weights before and after soaking into a 1 M VO_2_^+^ solution, respectively. *C*_VO_2_^+^_ is the initial V(V) concentration and *C*_VO^2+^_ is the V(IV) concentration obtained from the reduction of V(V).

#### 2.3.7 Membrane Characterization in VRFB

A VRFB single cell (C-Flow Lab 5 × 5) was built by sandwiching the membrane with two pieces of carbon felt electrodes (pretreated at 400 °C for 30 h) clamped by two graphite polar plates. The effective area of the cell was 25 cm^2^. Two solutions (40 and 80 mL), which consisted of 1 M VOSO_4_ in 2 M H_2_SO_4_, were used as an anolyte and a catholyte, respectively. These solutions were pumped into the cell at a flow rate of 20 mL min^–1^ cm^–2^. Nitrogen was bubbled through the anolyte to suppress undesired oxidation of vanadium (low oxidation states) by air, which leads to battery self-discharge.

Electrochemical measurements were conducted at room temperature in a Biologic potentiostat (potentiostat/galvanostat board e type for VSP3-e & VSP). The cutoff voltages were set at 0.9 and 2 V, respectively. The stability of SPES membranes was studied at 20 and 40 mA cm^–2^ for a long-term stability test.

The Coulombic efficiency (CE), voltage efficiency (VE), and energy efficiency (EE) were determined by using eqs 7–9, respectively, as follows^25^
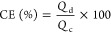
7
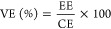
8
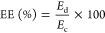
9where *d* and *c* denote the discharge and charge processes, respectively, *Q* is the capacity, *V* is the voltage, and *E* is the energy.

*In situ* membrane conductivity was measured by means of electrochemical impedance spectroscopy (EIS) using Solartron 1260 equipment with a Solartron 1287 electrochemical interface in a VRFB single cell previously described. Measurements were performed at an open-circuit voltage (OCV) in the frequency range between 10^–1^ and 10^6^ Hz, with an AC voltage of 10 mV.
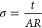
10where *t*, *A*, and *R* are the thickness of the membrane, the active area, and the total resistance of the cell, respectively.

Membranes were treated with a 2 M H_2_SO_4_ solution, and this solution was maintained in both cell compartments during the test. Membrane selectivity was calculated from the ratio between the ionic conductivity and vanadium ion permeability.

## 3 Results and Discussion

### 3.1 Properties of SPES Membranes: Strategy

**Scheme 2:**
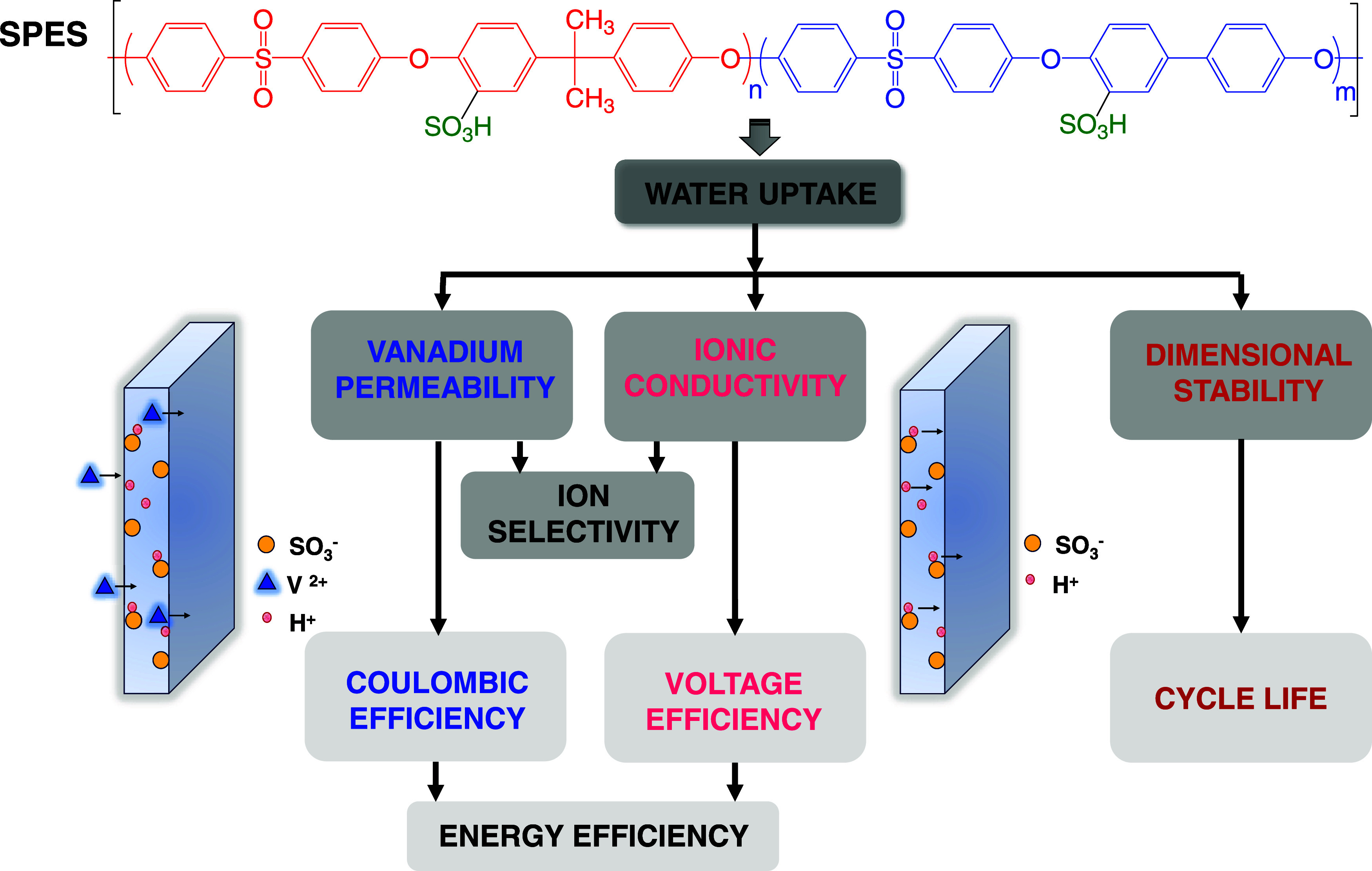
Relationship between the Efficiency of the Membranes on a VRFB (CE, VE, and EE) and Their Properties

The response of the PSU and PPSU blocks within the multiblock copolymers toward electrophilic aromatic substitution is clearly different. In particular, the PSU block is easily sulfonated, whereas the PPSU block reacts only with sulfonating agents under specific experimental conditions. Furthermore, increasing the proportion of the PSU block makes the sulfonation reaction more selective, achieving in some polymer matrices that only the PSU block is sulfonated. This behavior is useful for the design of materials with high performance as separators in VRFBs since it is intended to reach high values of ionic conductivity and low permeability to the active vanadium species. The first objective is achieved by increasing the number of functional groups, in this case, sulfonic groups, which provide high hydrophilicity to the membrane, and the second characteristic is achieved when the hydrophobicity of the membrane is high. A high value of ionic conductivity leads to a high voltaic efficiency. On the other hand, if the permeability of the membrane to the active species (crossover) is high, it would reduce the Coulombic efficiency. These opposing effects (hydrophilicity and hydrophobicity) are critical to successfully designing high-performance materials that perfectly match the demanding requirements of VRFBs. Therefore, it is necessary to find a compromise between both parameters by using a strategy of synthesis that allows to achieve high values of energy efficiency (defined as EE = VE × CE) to be able to propose these materials as separators in VRFBs (Scheme 2). To address this aim, in the present work, a family of SPES-based membranes were synthesized, characterized, and tested in a VRFB single cell.

### 3.2 Characterization of the Membranes

The membranes were obtained by a casting procedure from a solution of SPES, as described in Section 2.2. The degree of sulfonation (DS) of the two blocks was obtained from both ^1^H NMR analysis and ionic exchange capacity (IEC) values as described by Swaby et al.^23^ The DS of PSU varied from ∼0.8 to ∼2 (obtaining the highest value for the 60/40 copolymer), whereas a maximum value of DS around 0.8 for SPPSU was achieved for SPES 50/50. In the case of 60/40 and 75/25 copolymers, sulfonation occurred mainly in the block of PSU, leaving the PPSU portion almost unfunctionalized (DS_PPSU_ around 0.1 for both copolymers) due to the high selectivity in the sulfonation reaction on PES 60/40 and 75/25 copolymers as has been described by Swaby et al.^23^

#### 3.2.1 Water Uptake of Membranes in Acidic Solutions

The overall amount of absorbed water present in the swollen membranes influences both the total crossover of vanadium ions and the ionic conductivity values. Water uptake commonly leads to the dissociation of the ion pairs between the fixed ion-exchange group (SO_3_^–^) and solvated mobile ions.^29^Table 1 shows the WU_ac_% values for SPES membranes at 30 and 60 °C.

**Table 1:** WU_ac_% and Permeability of the SPES and Nafion Membranes

	WU_ac_%		
membrane	30 °C	60 °C	permeability × 10^7^ (cm^2^ min^–1^)
SPES 50/50	28 ± 4	31 ± 4	7.72 ± 0.01
SPES 60/40	27 ± 2	31 ± 2	2.03 ± 0.01
SPES 75/25	47 ± 4	50 ± 7	14.20 ± 0.02
Nafion 115	12 ± 2	18 ± 1	98 ± 6

As can be seen in Table 1, SPES 75/25 membranes show higher WU_ac_% (47% at 30 °C) than that obtained for SPES 50/50 and SPES 60/40 (28% at 30 °C). A similar behavior is also obtained at 60 °C, and the WU_ac_% values are slightly higher than those obtained at 30 °C. In general, it can be said that WU_ac_% values are slightly lower than those of WU% determined for the same series of membranes,^23^ in agreement with the information reported in the bibliography.^30,31^ The series of membranes synthesized exhibit low water uptake, which favors the dimensional stability and consequently longer cycle life on the VRFB.

The swelling degree (SD_Thickness_%), as well as the variation in the area (SD_Area_%) of the SPES 50/50, 60/40, and 75/25 membranes, have been determined after being soaked in a 2 M H_2_SO_4_ solution during 48 h at 30 °C. The values obtained are shown in Table S1. SPES 50/50 and 60/40 membranes exhibit values for SD_Thickness_% (26 ± 9 and 16 ± 1, respectively) and SD_Area_% (18 ± 2 and 18 ± 8, respectively) that are significantly equal, as was observed with the WU_ac_% (28 ± 4 and 27 ± 2 for SPES 50/50 and SPES 60/40 membranes, respectively). In the case of the membrane with a higher proportion of the polysulfone block, SPES 75/25, the SD_Thickness_% value follows the same tendency as WU% and WU_ac_%. However, the SD_Area_% could not be accurately determined due to the high WU% obtained. Therefore, when these membranes are hydrated, not only does their thickness increase but also does their area.

#### 3.2.2 Membrane Permeability to Vanadium Ions

Permeability of the membranes to vanadium ions is a crucial parameter used to evaluate the capacity loss during the charge/discharge cycles in a VRFB and, consequently, its long-term operation.^11^ The crossover of vanadium ions through the membrane produces efficiency and capacity losses, which leads to a reduction in the long-term stability and cyclability of the battery. Furthermore, in other types of RFBs, it causes the mixing of anolyte and catholyte, causing irreversible contamination. So, the IEMs used as an electric insulator and liquid separator must conduct protons and avoid the crossover of vanadium ions.

**Figure 1:**
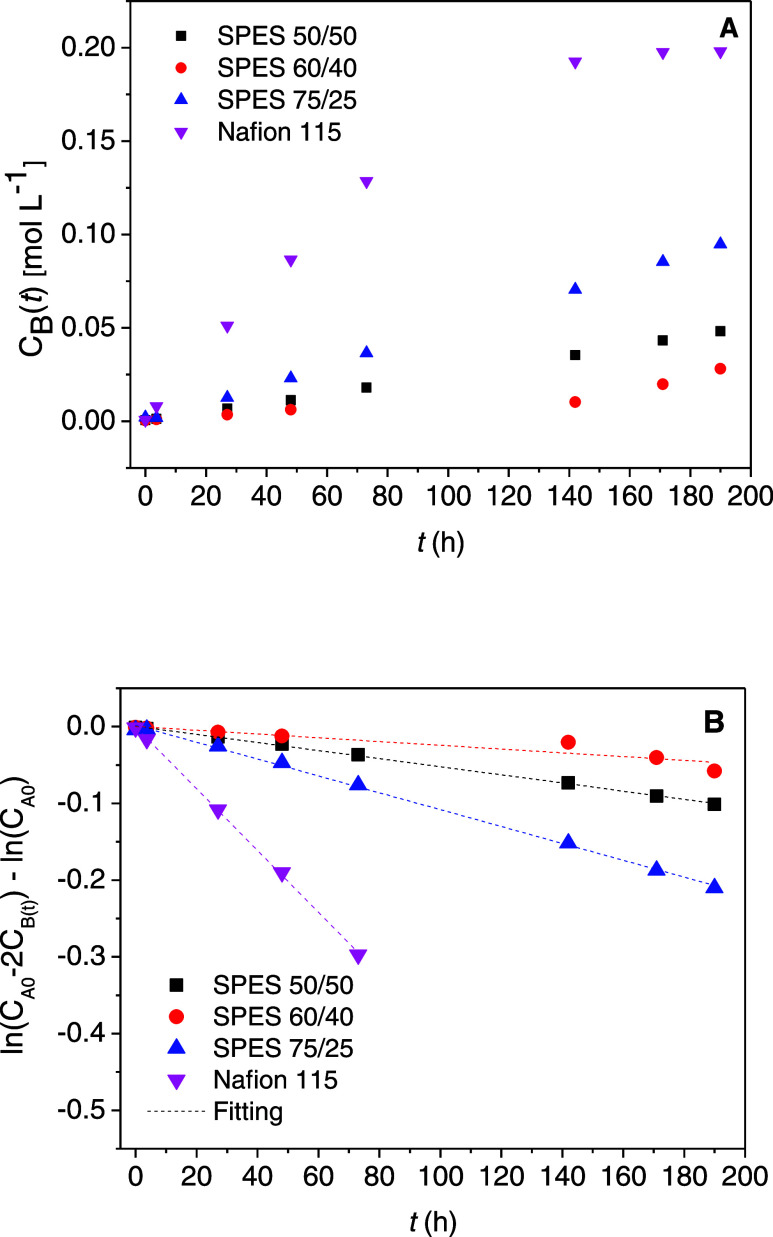
(A) Evolution of vanadium(IV) concentration in compartment B with time for SPES and Nafion 115 membranes. (B) ln(*C*_A0_ – 2*C*_B_(*t*)) – ln(*C*_A0_)–*t* plots for SPES and Nafion 115 membranes.

The vanadium ion crossover rate was determined by using a homemade cell with two reservoirs, as explained in the Section 2. The evolution of the VO^2+^ concentration in the vanadium-deficient compartment with time for SPES and Nafion 115 membranes is shown in Figure 1A.

After almost 200 h (more than 8 days), the concentration of VO^2+^ that has crossed the membrane, measured in the V-deficient compartment, is linearly proportional to the time according to the following sequence: Nafion 115 > SPES 75/25 > SPES 50/50 > SPES 60/40. Therefore, SPES membranes show a lower permeability to vanadium ions than the commercial Nafion 115 one, and this difference is much more pronounced than that established between the SPES membranes themselves since they all present very similar behavior.

As shown in eq 4 of the previous section, the vanadium ion permeability of these IEMs can be easily obtained from the slope of ln(*C*_A0_ – 2*C*_B_(*t*)) – ln(*C*_A0_) *vst* plot (Figure 1B). Table 1 shows the permeability values obtained for SPES and the Nafion 115 membranes. The SPES 75/25 membrane shows higher permeability (14.2 × 10^–7^ cm^2^ min^–1^) than those observed for SPES 50/50 (7.72 × 10^–7^ cm^2^ min^–1^) and 60/40 (2.03 × 10^–7^ cm^2^ min^–1^). This behavior can be explained in terms of water absorption. The membrane with the highest WU_ac_% (SPES 75/25) shows the greatest vanadium ion permeability. However, the SPES 50/50 and SPES 60/40 membranes have lower and very similar WU_ac_% values, resulting in smaller and very close permeability values.

All of these SPES membranes show lower permeability than the commercial ones, such as Nafion 115 (98 × 10^–7^ cm^2^ min^–1^). All of these values are in the same order of magnitude as those reported for similar membranes. Thus, sPP-*b*-PES and sPP-*b*-SPK copolymers show a permeability of 2.60 × 10^–7^ and 2.50 × 10^–7^ cm^2^ min^–1^, respectively, while sPP-*b*-PPSU and sPP-*b*-PPES copolymers exhibit slightly higher values of 5.80 × 10^–7^ and 4.50 × 10^–7^ cm^2^ min^–1^, respectively.^20^

#### 3.2.3 Mechanical Properties

The mechanical behavior of SPES membranes was studied before and after treatment in a 1 M VO^2+^ solution for 27 days. This study aims to evaluate the effect that exposure to highly concentrated corrosive species H_2_SO_4_ and VO^2+^ has on the membrane’s mechanical properties. Table 2 shows the tensile strength (TS) and strain values for SPES membranes before and after their treatment in a 1 M VOSO_4_ solution for 27 days. In SPES 60/40 and 75/25 membranes, the tensile strength does not change significantly when the membranes are immersed in an acidic vanadium solution. The same does not happen with the SPES 50/50 membrane. When dry, the tensile strength is very high and decreases by approximately 30% when wet (with a water and vanadium solution). In these membranes, the sulfonic groups are homogeneously distributed between the two blocks of the copolymer (PSU and PPSU), and the plasticizing effect of water is very pronounced.^32^ Nevertheless, in all SPES membranes, the TS is maintained above ∼45 MPa after being treated with the vanadium solution (Figure S4). The TS of SPES membranes is clearly higher than that observed for the commercial membranes used successfully in these electrochemical systems, such as Nafion 115. So, the SPES membranes described in this work can maintain their mechanical stability under the operation conditions used in VRFBs.

**Table 2:** Tensile Strength and Strain of the Membranes before and after Their Treatment in a 1 M VOSO_4_ Solution for 27 Days

	tensile strength (MPa)		strain (%)	
membrane	before V(IV)	after V (IV)	before V(IV)	after V (IV)
SPES 50/50	87 ± 8	58 ± 9	3.7 ± 0.4	6 ± 2
SPES 60/40	51 ± 3	46 ± 4	1.6 ± 0.1	5 ± 1
SPES 75/25	53 ± 5	54 ± 5	2.8 ± 0.5	7 ± 1
Nafion 115	24 ± 1	26 ± 4	65 ± 5	80 ± 9

On the other hand, strain limits of IEMs increased after their treatment with the vanadium solution. The VO^2+^ solution seems to act as a plasticizer for the membrane structure. In general, SPES membranes exhibit higher mechanical properties than those of Nafion 115. The values obtained here are like those found in the literature for this kind of polymer.^32,33^

#### 3.2.4 *Ex Situ* Chemical Stability

The chemical stability of a polymer electrolyte membrane indicates its capability to withstand the extreme chemical environment within a cell of a battery system. The degradation of the membrane due to severe oxidation by VO_2_^+^ ions in the electrolyte can also result in poor stability for both the membrane and the battery.^34^

To assess the chemical stability of SPES membranes, an *ex situ* evaluation was conducted using the mass loss (measured in wt %) observed in the membrane after exposure to a 1 M VO_2_^+^ solution for 56 days at 40 °C. Additionally, the changes in VO^2+^ concentration resulting from the reduction of VO_2_^+^ were monitored. These assessments were carried out under accelerated conditions, simulating a fully charged battery status. Table S2 shows the results of the test.

The SPES 60/40 membrane exhibits the lowest stability in the VO_2_^+^ solution. It shows a reduction of VO_2_^+^ by 3.8% and a mass loss of 23.8%. These values are comparatively higher than those observed for the SPES 50/50 and 75/25 membranes. Among these, the SPES 75/25 membrane demonstrates the highest stability with a mass loss of 9% and a VO_2_^+^ reduction of 1.5%. These values are similar to those reported for Nafion membranes (1% of VO_2_^+^ to VO^2+^ after 21 days) studied by Teng et al.^35^ Zhang et al.^36^ determined the mass loss for SPEEK (DS = 87%) and SPSU (DS = 76%) obtaining values of 3.8 and 4.7%, respectively. The reduction of VO_2_^+^ to VO^2+^ after 21 days in 1.5 M VO_2_^+^ solution was also studied, and the obtained values were 6.1 and 3.5% for SPEEK (DS = 87%) and SPSU (DS = 76%), respectively.

The behavior of these IEMs in terms of chemical stability could be attributed to the arrangement of the sulfonic groups within the copolymer structure. While it is reported in the literature that a higher DS in SPSU homopolymer (highly sulfonated) promotes the matrix oxidation and the backbone degradation,^36^ the distribution of the sulfonic groups and the high molecular weight of SPES copolymers increase the resistance on the VO_2_^+^ solution. This feature is evident in the case of the SPES 75/25 copolymer.

**Figure 2:**
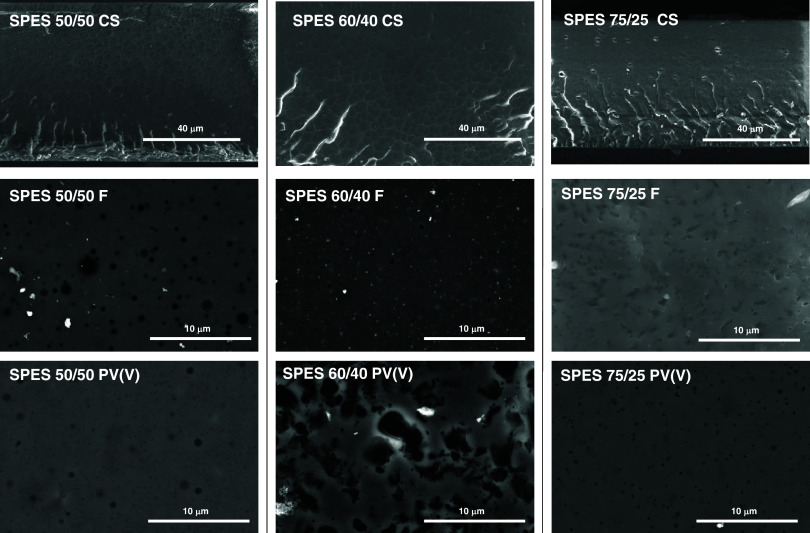
SEM images of the cross-section (CS) and surface (F) of SPES membranes before (top) and after the (PV) V (bottom) stability test.

Morphology of SPES membranes was also evaluated before and after the stability test. Figure 2 shows the SEM images of both the cross-section (CS) and the surface of the membranes.

SEM images of SPES membranes show a homogeneous morphology before the stability test, as can be seen in the cross-section images on top; only in SPES 75/25 are some holes with 400 nm of diameter observed due to the solvent evaporation process. The images of the membranes after being treated in a 1 M VO_2_^+^ solution show no significant sign of degradation. Nevertheless, it was found that the SPES 60/40 membrane showed slight chemical degradation by the observation of certain holes (black spots) in the surface. The chemical stability of the membranes can be associated with the high molecular weight of these materials, 35, 16, and 71 kDa, for SPES 50/50, SPES 60/40, and SPES 75/25, respectively.^23^ SPES 60/40 showed lower molecular weight, and this fact could be used to explain its lower chemical stability.^22^

### 3.3 VRFB Single-Cell Performance

While all of the performance evaluation parameters discussed in Section 3.2 allow the comprehensive characterization of the properties of the SPES copolymer membranes, it is also of vital importance to evaluate the ultimate performance of membranes when used in a flow battery single cell. Therefore, to evaluate *in situ* membrane performance, results for ionic conductivity and charge–discharge cycling experiments are depicted in this section.

#### 3.4.1 *In Situ* Ionic Conductivity of SPES Membranes

Figure S5 shows the Nyquist plots obtained for the synthesized membranes. All tested membranes show a similar shape of the impedance response. A high-frequency intersection with the real axis can be seen, which represents the ohmic resistance of the system. At lower frequencies, two flattened semicircles corresponding with the membrane and electrolyte (left/high frequency) and electrode and electrolyte (right/low frequency) interfacial resistances associated with charge transfer and mass transfer processes through the carbon felt electrodes and the membrane.^37−40^ The area specific resistance (ASR) considers the active area (1 cm^2^) and the total resistance of the VRFB cell that was obtained from the Nyquist plot. A higher ASR is observed for SPES 60/40 and SPES 75/25 membranes than for SPES 50/50 and Nafion 115. The ionic conductivity is also evaluated in 0.5, 1, and 2 M H_2_SO_4_ solutions at RT (Figure S6A–D, respectively). The ionic conductivity increases when the H_2_SO_4_ concentration increases. This trend is observed in similar membranes tested with other electrolytes such as deionized water,^10,35,41,42^ sulfuric acid, and vanadium electrolyte (3.5 M H_2_SO_4_ and 2 M VOSO_4_).

Table 3 shows the values of ASR of the membranes measured in a 2 M H_2_SO_4_ solution at RT. It is observed that the membranes exhibit low ASR at RT, which is expected in these membranes as obtained for *ex situ* measurements in our previous work at 30 °C (0.06 and 0.5 mS cm^–1^ for SPES 60/40 and SPES 75/25, respectively).^23^ The SPES 50/50 membrane slightly sticks out with ∼60% higher compared to SPES 60/40 and SPES 75/25. These values of ionic conductivity are key indicators of the performance of the single cell because high ionic conductivity results in high voltage efficiency. The copolymers are slightly different in terms of IEC, but the distribution of sulfonic groups is the main difference among them. In SPES 50/50 membranes, the sulfonic groups are homogeneously distributed between two blocks, whereas in SPES 60/40 and 75/25 membranes, sulfonic groups are located in PSU blocks.

**Table 3:** Area-Specific Resistance, Thickness, and *In Situ* Ionic Conductivity of the SPES Membranes Measured in a 2 M H_2_SO_4_ Solution at RT

membrane	ASR (Ω cm^2^)	thickness (μm)	*in situ* ionic conductivity (mS cm^–1^)
SPES 50/50	3.95 ± 0.04	53	1.34 ± 0.05
SPES 60/40	3.803 ± 0.008	30	0.78 ± 0.01
SPES 75/25	5.8 ± 0.5	47	0.810 ± 0.06
Nafion 115	4.56 ± 0.07	127	2.79 ± 0.08

#### 3.4.2 Ionic Selectivity of the Membranes

The ion selectivity of SPES membranes is defined as the ratio of the proton conductivity to VO^2+^ permeability. The values obtained for SPES 50/50, SPES 60/40, and SPES 75/25 were 1.74 × 10^6^, 3.84 × 10^6^, and 0.57 × 10^6^ S min cm^–3^, respectively. SPES 50/50 and 60/40 membranes show the highest values of ionic selectivity. Zhang et al.^36^ reported values of selectivity for SPSU (DS = 62%) of 4.6 × 10^3^ S min cm^–3^. So, SPES 50/50, in view of its previous characterization (WU_ac_%, mechanical stability, and ion selectivity), seems to show adequate properties to be used as a separator in the VRFB single cell.

#### 3.4.3 Charge–Discharge Performance

**Figure 3:**
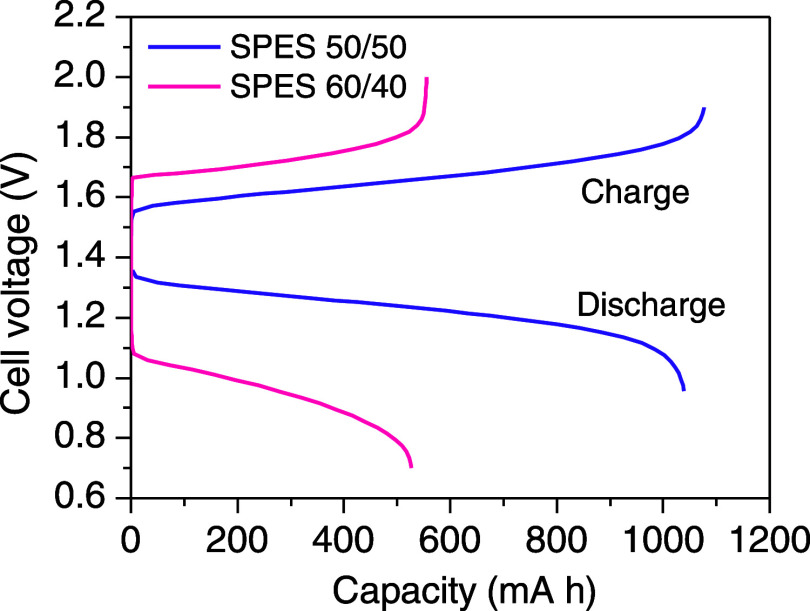
Charge–discharge profile of VRFB with SPES 50/50 and SPES 60/40. A current density of 20 mA cm^–2^ and 1 M VOSO_4_ in a 2 M H_2_SO_4_ solution.

In order to test the suitability of using these membranes as separators in VRFB, charge–discharge experiments were performed to examine the cell performance for energy storage and power generation under operating conditions. Figure 3 shows the comparison of a charge–discharge cycle in a VRFB single cell for a voltage range between 0.9 and 2 V and at a current density of 20 mA cm^–2^ for membranes prepared with SPES 50/50 and SPES 60/40 (Figure S7). The SPES 75/25 membrane could not be tested under these conditions, as the polarization was too high.

The battery shows similar charge and discharge values given by the concentration of vanadium in the electrolyte. In the case of SPES 50/50, charge and discharge capacities are very close, which indicates that the membrane shows high Coulombic efficiency (Figure 3). This is not the case for the membrane prepared with SPES 60/40, as the polarization was so high (due to a very poor voltage efficiency) that maximum capacity could not be reached for this battery in charge, and an even lower discharge capacity was obtained in the voltage range tested.

**Figure 4:**
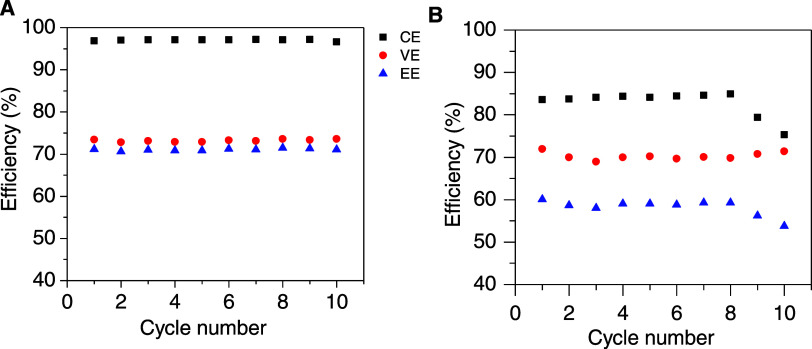
Coulombic, Voltage, and Energy efficiencies for 10 cycles of the membrane (A) SPES 50/50 and (B) Nafion 115 at a current density of 20 mA cm^–2^.

The CE of the VRFB reaches a value of nearly 97%. Figure 4A displays the fluctuation of CE, VE, and EE during the charge–discharge cycles. No fading of these values for achieved efficiencies has been observed. The VE of the VRFB with this membrane is ∼74%, which is similar to that obtained for Nafion 115 (Figure 4B and Table 4) and Nafion 212 (Table 4) measured under the same experimental conditions. These values are also comparable with other results obtained in the literature for polymeric membranes such as Fumasep 930,^43^ SPEEK,^44^ and cross-linked sulfonated poly(phenylene sulfide sulfone) (sPSSc 19)^11^ (Table 4).

**Table 4:** Coulombic, Energy, and Voltage Efficiencies of PEMs for VRFBs

membrane	reference	CE (%)	VE (%)	EE (%)
SPES 50/50	this paper	97.6 ± 0.1	72.8 ± 0.2	71.0 ± 0.2
Nafion 212	this paper	76 ± 1	68.5 ± 0.5	51.9 ± 0.4
Nafion 115	this paper	83 ± 3	70.3 ± 0.8	60 ± 2
Fumasep 930	(43)	98	82	80
SPEEK	(44)	94	82	77
sPSSc 19	(11)	92	88	81

This result is related to the high ionic conductivity of this membrane, while this parameter decreased significantly when the proportion of PPSU in the polymer was reduced, resulting in a very low VE and a huge polarization, as commented on previously. Thus, the EE for the SPES 50/50 membrane resulted in 71%. Finally, the long-term stability of the membrane was tested by obtaining up to 200 charge–discharge cycles at 40 mA cm^–2^ and analyzing the variation in Coulombic efficiency of the battery (Figure S8), observing a stable behavior with no significant CE variation along the cycles. Figure S9 shows the Coulombic, energy, and voltage efficiency obtained for SPES 50/50 at different current densities (60, 80, 100, and 120 mA cm^–2^).

The CE, VE, and EE values for the battery assembled with the SPES 50/50 membrane are close to those obtained in the same conditions with Nafion 115 and other polymers proposed for this application, as shown in Table 4. Another advantage of these types of materials is their low cost. The synthesis of 10 g of SPES copolymer costs 40 euros, and with this amount of polymer, a 40 cm × 40 cm membrane can be prepared. Nafion 115 membranes, 30 cm × 30 cm, are ten times more expensive than SPES membranes. So, these membranes seem to be suitable candidates to be used as separators in VRFBs.

## 4 Conclusions

This study focused on exploring the potential of nonfluorinated polymers, specifically block copolymers based on polysulfone (PSU) and polyphenylsulfone (PPSU), as promising candidates for ion-exchange membranes in vanadium redox flow batteries, with the aim of replacing Nafion. A series of copolymers based on sulfonated polysulfones with different PSU/PPSU ratios were prepared and used as precursors of ion-exchange membranes. The distribution of sulfonic groups within the copolymer matrix was found to significantly affect the membrane behavior. So, with the increasing amount (in mol %) of PPSU in the copolymer from 25 or 40 to 50%, sulfonation becomes nonselective, allowing the introduction of sulfonic groups not only in PSU units but also in PPSU ones. SPPSU could not be further increased due to the lower reactivity of the structure to the sulfonation reaction. This homogeneous distribution of sulfonic groups along the polymeric backbone, found in the case of 50/50 copolymer, improves the key properties of the polymer for this application, *i.e.*, higher ionic conductivity, lower permeability to ionic vanadium species, and, thus, better selectivity. These two factors increase the Coulombic efficiency and, mainly, the voltage efficiency of the VRFB along the series, passing from a VRFB that could not be cycled for the SPSU/SPPSU 75/25 membrane to one with high polarization (poor VE) for 60/40. In the end, we are able to obtain excellent results in terms of efficiency and long-term stability for SPSU/SPPSU 50/50. Consequently, PSU/PPSU 50/50 copolymer membrane stands out, demonstrating performance similar to, or even better than, commercial Nafion membranes and other nonfluorinated polymers proposed in the previous literature.

This work successfully demonstrated the possibility of using polysulfone-based copolymers as effective ion-exchange membranes in the VRFB. Ionic conductivity, mechanical properties, and chemical stability can be tuned to improve their performance, as shown here. Furthermore, they offer other possibilities that can be explored in future work to improve their performance, such as further functionalization with other active species, use of cross-linking agents, or modifying the processing to optimize the micro- and macrostructure of the final product. These results highlight the versatility of these materials.

## Supporting Information Available

Additional experimental details and results obtained in the characterization of the materials such as ^1^H NMR analysis; chemical stability in a VO_2_^+^/H_2_SO_4_ medium; mechanical and electrochemical behavior; and battery single-cell performance (PDF)

## Abbreviations

RFBs: redox flow batteries
VRFBs: vanadium redox flow batteries
IEM: ion-exchange membrane
PP: polypropylene
SPEEK: sulfonated polyether ether ketone
SPES: sulfonated polyether sulfone
PSU: polysulfone
PPSU: polyphenylsulfone
CE: Coulombic efficiency
VE: voltage efficiency
EE: energy efficiency
BP: 4,4′-dihydroxybiphenyl
DFDPS: 4,4′-difluoro-diphenylsulfone
BPA: 4,4′-isopropylidenediphenol
TMSCS: trimethylsilyl chlorosulfonate
CDCl_3_: chloroform
H_2_SO_4_: sulfuric acid
DMF: dimethylformamide
DCE: 1,2-dichloroethane
DMAc: *N*,*N*-dimethylacetamide
DMSO: dimethyl sulfoxide
TMS: tetramethylsilane
TGA: thermogravimetric analysis
FE SEM: field emission scanning electron microscopy
WU: water uptake
DMA: dynamo mechanical analysis
OCV: open-circuit voltage
DS: degree of sulfonation
EIS: electrochemical impedance spectroscopy
^1^H NMR: proton nuclear magnetic resonance
IEC: ionic exchange capacity
ASR: area specific resistance
TS: tensile strength
sPSSc: cross-linked sulfonated polyphenylene sulfide sulfone
