# Supplementary material for: Block Copolymer-Based Membranes for Vanadium Redox Flow Batteries: Synthesis, Characterization, and Performance
Source: ACS Appl Polym Mater. 2024 Jul 29;6(15):8966–76. doi: 10.1021/acsapm.4c01262 (PMC11320381; doi:10.1021/acsapm.4c01262)
Supplement: Supplementary file 1 — ap4c01262_si_001.pdf [file ap4c01262_si_001.pdf]

Supporting Information

Block Copolymer-Based Membranes for Vanadium Redox Flow Batteries: Synthesis, Characterization and Performance

Sydonne Swaby [a], Diego Monzón [a], Nieves Ureña [a], José Vivo Vilches [a], Jean-Yves Sanchez [a,b,c], Cristina Iojoiu [b,c] Alejandro Várez [a], María Teresa Pérez-Prior [a], Belén Levenfeld [a]*

[a] *Universidad Carlos III de Madrid. Departamento de Ciencia e Ingeniería de Materiales e Ingeniería Química, IAAB. Avda. Universidad, 30, 28911 Leganés, Madrid, Spain.*

[b] *University Grenoble Alpes, LEPMI, 38000 Grenoble, France.*

[c] *CNRS, LEPMI, 38000 Grenoble, France*

**Corresponding author. Tel.: +34 916 249 849*

E-mail address: maperezp@ing.uc3m.es

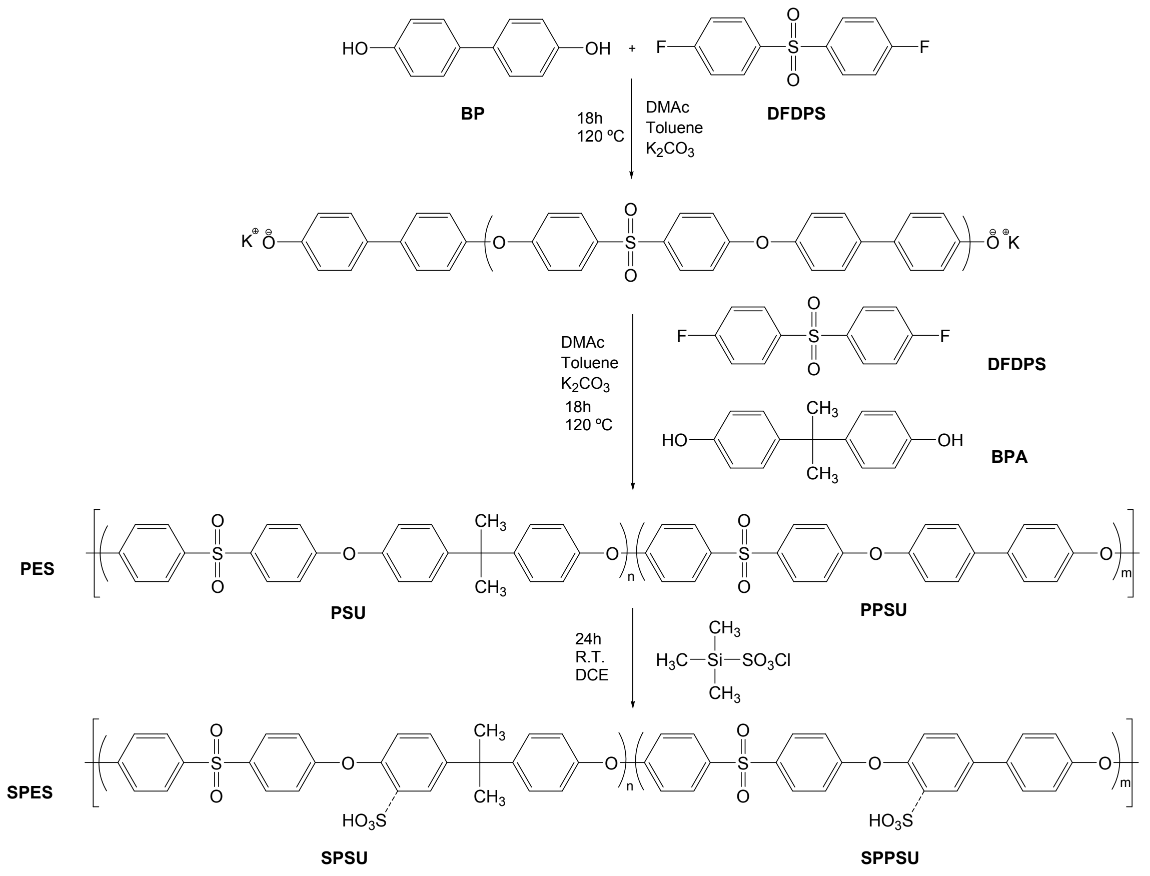

Figure S1. Synthesis of SPES copolymers.

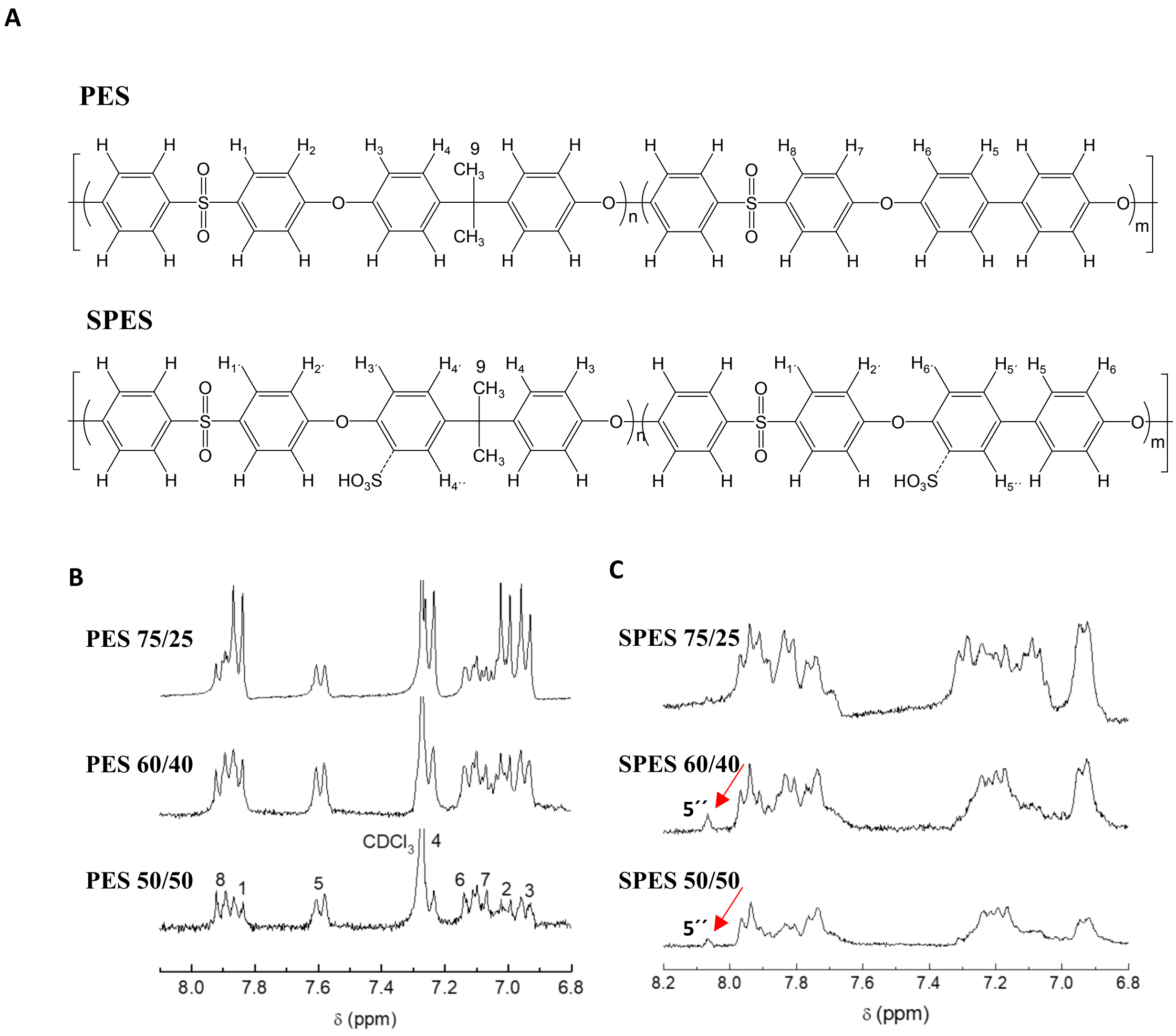

Figure S2. Chemical structures of PES and SPES (A). ^{1}H-NMR spectra of PES (B) and SPES (C) at different PSU/PPSU proportions. $DS_{PPSU} = (6 \cdot I(H_{5''}))/(I(H_9 \cdot m/n))$; The DS of the PSU segment is also estimated from IEC values by subtracting the DS from PPSU units. The IEC is measured by means of standard titration, as described in Ureña, N. *et al* [1].

Table S1. Swelling degree values of SPES membranes.

Membrane	$SD_{Thickness}\%$	$SD_{Area}\%$
SPES 50/50	26 ± 9	18 ± 2
SPES 60/40	16 ± 1	18 ± 8
SPES 75/25	30 ± 6	-

Table S2. Mass loss, and reduction of VO_2^+ of SPES membranes after the chemical stability test (1 M VO_2^+, 56 days, 40 °C).

Membrane	% Mass loss	% Reduction of VO_2^+
SPES 50/50	18 ± 3	2.5 ± 0.3
SPES 60/40	23.8 ± 0.3	3.8 ± 0.4
SPES 75/25	9 ± 1	1.5 ± 0.2

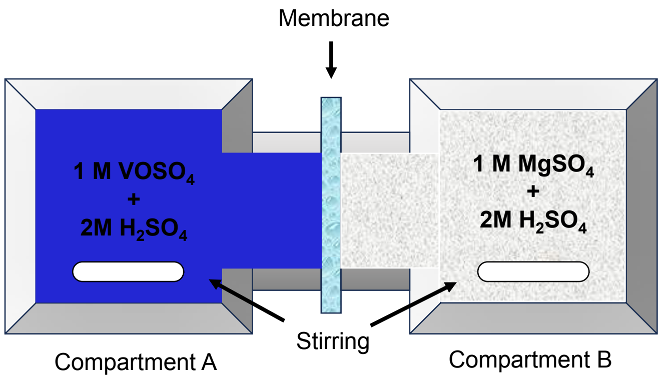

Figure S3. Schematic configuration of two compartments cell used for vanadium ions permeability measurements.

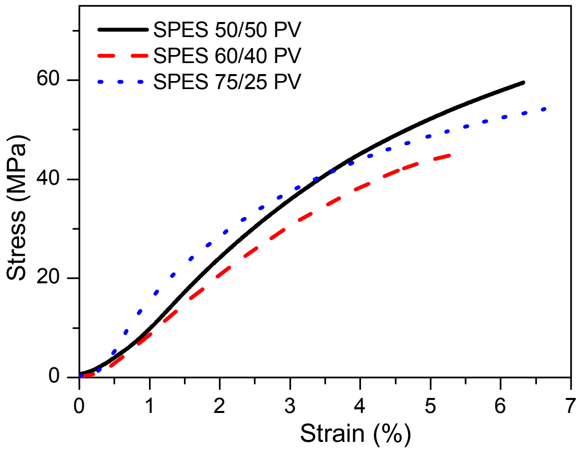

Figure S4. Stress-strain curves of the membranes SPES 50/50, SPES 60/40 and SPES 75/25 after vanadium permeability tests.

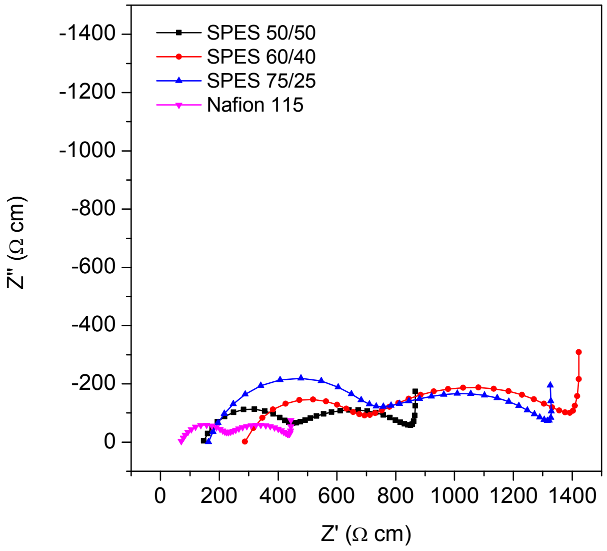

Figure S5. Nyquist representation of SPES membranes in a 2 M H_2SO_4 solution.

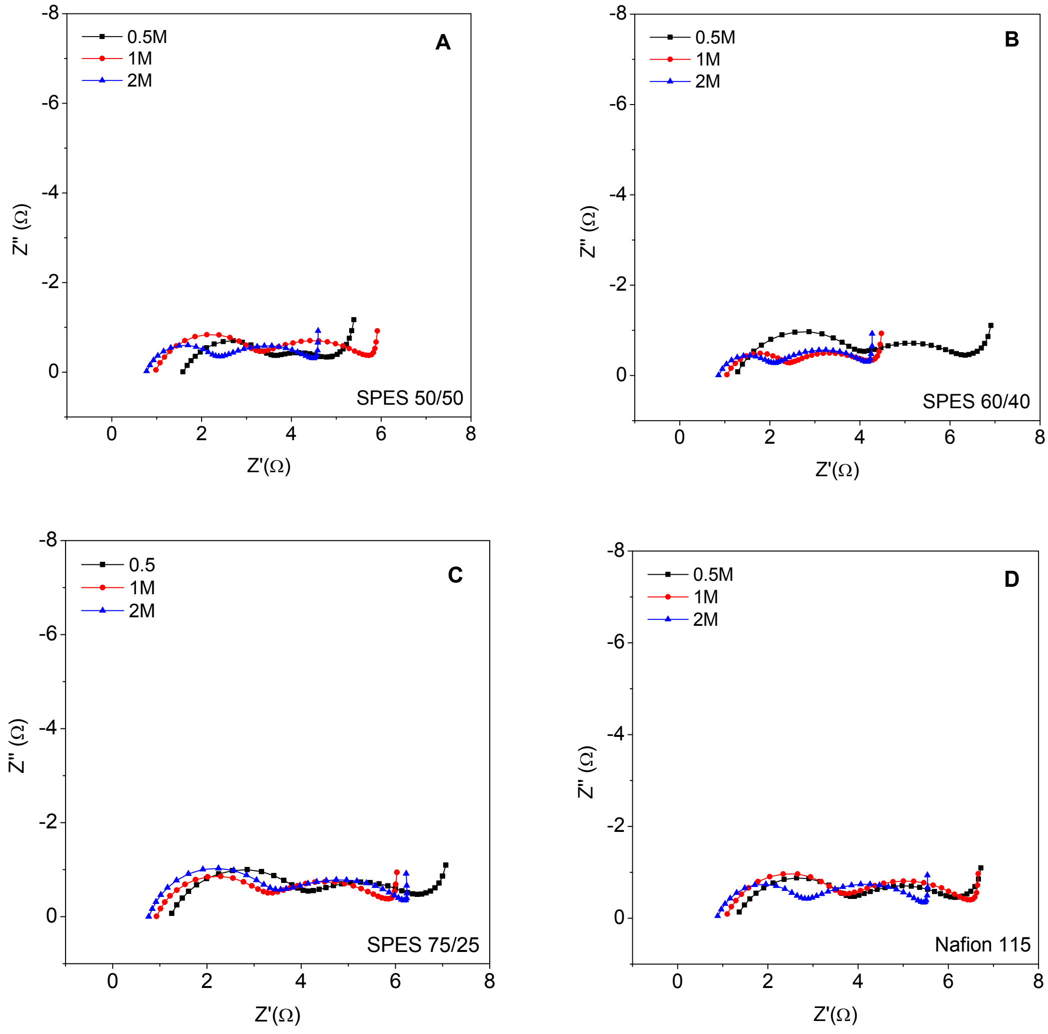

Figure S6. Nyquist representation of the membrane (A) SPES 50/50, (B) SPES 60/40, (C) SPES 75/25, (D) Nafion® 115 at different H_2SO_4 concentrations (0.5, 1, and 2 M).

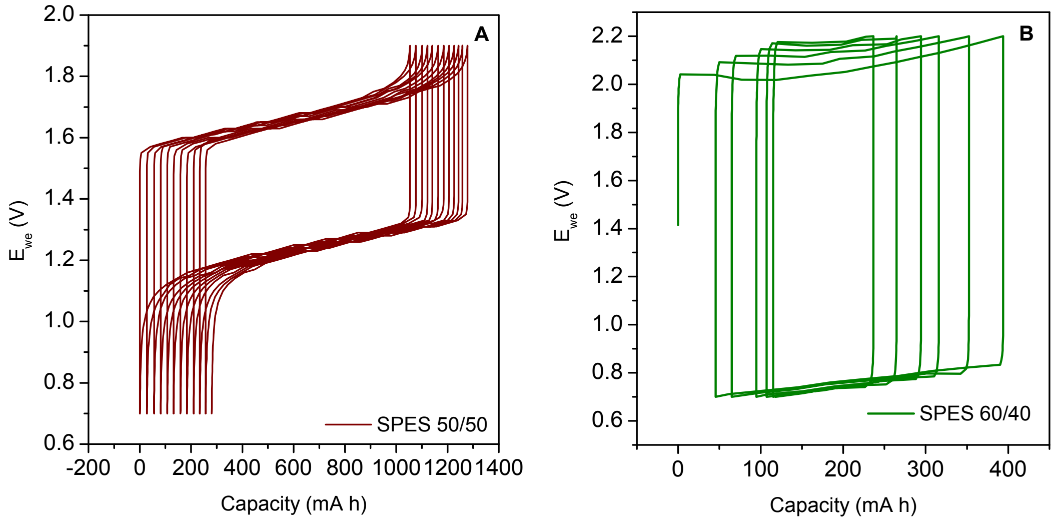

Figure S7. Charge-discharge curves of (A) SPES 50/50 and (B) SPES 60/40 at R.T. and current density 20 mA cm^{-2} in 1 M $VOSO_4$ and 2 M H_2SO_4.

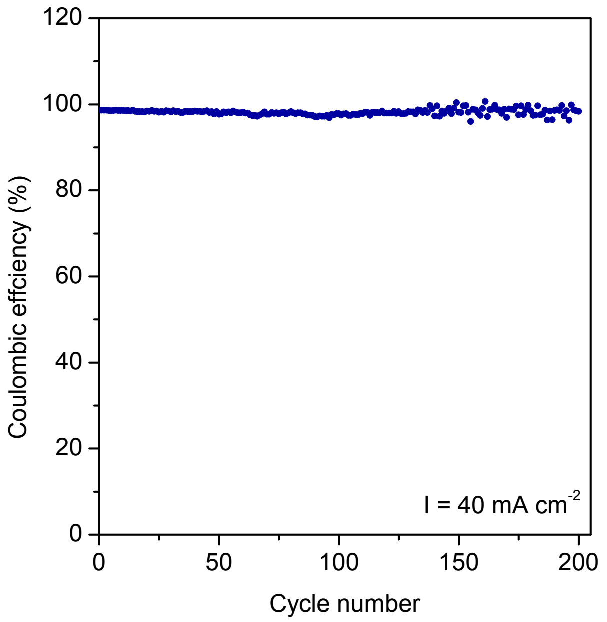

Figure S8. Charge-discharge curves of SPES 50/50 at R.T. and a current density of 40 mA cm^{-2} in 1 M $VOSO_4$ and 2 M H_2SO_4 for 200 cycles.

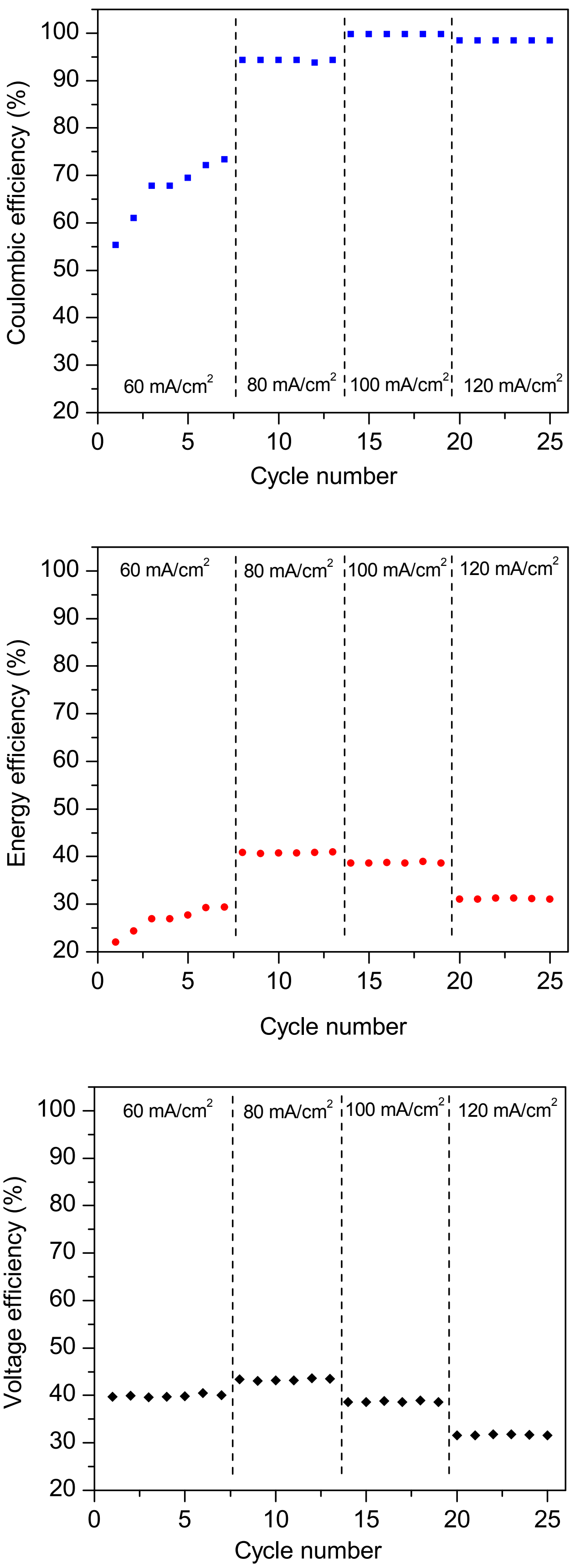

Figure S9. Coulombic, energy and voltage efficiency of SPES 50/50 membrane at different current density (60, 80, 100 and 120 mA cm^{-2}).

References

[1] Ureña, N.; Pérez-Prior, M. T.; Río, C. del; Várez, A.; Sanchez, J.-Y.; Iojoiu, C.; Levenfeld, B. Multiblock Copolymers of Sulfonated PSU/PPSU Poly(Ether Sulfone)s as Solid Electrolytes for Proton Exchange Membrane Fuel Cells. Electrochim Acta 2019, 302, 428–440. https://doi.org/10.1016/j.electacta.2019.01.112.
